# Prolactin Monitoring in Community Patients Receiving Long-Acting Injectables

**DOI:** 10.1192/bjo.2026.11828

**Published:** 2026-06-30

**Authors:** Rachel Rajaratnam, Basavaraja Papanna

**Affiliations:** Essex Partnership University NHS Foundation Trust, Colchester, United Kingdom

## Abstract

**Aims:**

The purpose of this audit was to ensure that patients who are receiving long acting injectables of antipsychotic medication are having their prolactin monitored as per national and Trust guidelines.

Prolactin is a hormone responsible for lactation, breast development, and other actions needed to maintain homeostasis. Hyperprolactinemia is defined as an increase in prolactin level above the normal range that is sustained. The Trust guidelines outline that a baseline prolactin must be established before starting or changing an antipsychotic. The patient must then be evaluated for symptoms of hyperprolactinemia and prolactin serum level re-checked 3 months later.

**Methods:**

This was a retrospective review using the serum prolactin levels available on ICE test results system and information available from clinics letters and documentation. All patients were above the age of 18 and receiving regular depots at the community depot clinic. All patients presented to the depot clinic between the 1^st^ May 2024 and 31^st^ May 2024.

A study sample was obtained using a list of patients from the depot clinic. Out of that list, 108 patients present to the depot clinic within the set timeframe. 31 patients were excluded as they were on an Aripiprazole depot, which does not requiring monitoring of prolactin levels as per NICE and Trust guidelines. A further 18 patients were excluded as the depot was started before 2014, and a start date was unobtainable. 2 patients started the depot in a different Trust and 2 patients started the depot in South Essex, therefore a baseline prolactin was unobtainable. A total of 55 patients were finally included in the study sample.

The target for compliance was 100%.

**Results:**

Of the 55 patients commenced on long-acting injectable treatment, 33 (60%) did not have a baseline prolactin level measured prior to initiation, and 22 (40%) did not have prolactin levels checked within the preceding year. Sixteen patients (29%) had neither a baseline nor a follow-up prolactin measurement. Physical health checks within the last year were completed and documented in 37 patients (67%). Overall compliance was 40% for baseline prolactin monitoring, 60% for annual prolactin monitoring, and 67% for annual physical health checks.

**Conclusion:**

There was inadequate monitoring of prolactin levels in community patients receiving long acting injectables, as the target for compliance was not met. As need for monitoring needs to be highlighted, the results of this audit were presented to the community team and at the local teaching.

